# Immunosuppressive regimens on conversion of cytomegalovirus infection to disease in liver transplant recipients

**DOI:** 10.22088/cjim.13.4.721

**Published:** 2022

**Authors:** Amirhassan Rabbani, Shiva Aliabbar, Saman Nikeghbalian, Ali Malek-hosseini, Mana Baziboroun

**Affiliations:** 1 Taleghani Hospital, Department of Transplant and Hepatobiliary Surgery, Shahid Beheshti University of Medical Sciences, Tehran, Iran; 2 Shiraz Transplant Center, Abu-Ali Sina Hospital, Shiraz University of Medical Sciences, Shiraz, Iran; 3 Infectious Disease and Tropical Medicine Research Center, Shahid Beheshti University of Medical Sciences, Tehran, Iran; 4 Cancer Research Center, Health Research Institue, Babol University of Medical Sciences, Babol, Iran

**Keywords:** Cytomegalovirus, Liver transplant, Immunosuppression

## Abstract

**Background:**

Cytomegalovirus (CMV) disease is one of the most common infectious complications after liver transplantation. It is the cause of numerous morbidity and mortalities. Intensity of immunosuppression defined as overall immunosuppressive drug dosage seems to affect infectious complications. The main purpose of this study is to investigate the intensity of immunosuppression on conversion of CMV infection to disease in this population.

**Methods:**

In this cross-sectional study, we retrospectively evaluated and analyzed the data of all recipients who underwent orthotopic liver transplantation (OLT) between March 2014 and March 2016 and had positive serum PCR for CMV after transplantation in follow- up course. Of 134 recipients, only 66 adult liver transplant recipients were eligible to be studied. Multiple variables such as MELD score, cold ischemic time, warm ischemic time, operative data, immunosuppressive drugs and regimen, plasma CMV viral load, donor and recipient CMV IgG serostatus were recorded and analyzed.

**Results:**

of the 66 patients, 50 (76%) had CMV infection and 16 (24%) had disease. There was significant association between donor CMV IgG serostatus, extra corticosteroid pulse therapy, acute cellular rejection, serum tacrolimus level and conversion of CMV infection to CMV disease (P=0.005, 0.001, 0.031, 0.031).

**Conclusion:**

It seems that the intensity of immunosuppression has influence on conversion rate of CMV infection to disease in liver recipients.

Cytomegalovirus is one of the most common infectious complications after liver transplantation (1). It usually occurs 3 months after operation (2). CMV disease constitutes wide range of symptoms from a viral infection to tissue invasive disease (3). Multiple risk factors affect CMV disease (4, 5).Donor and recipient CMV IgG serostatus at the time of transplantation have effect. CMV D+/R- has the highest risk for disease (6). Intensity of immunosuppression especially anti-lymphocyte antibody drugs has influence (7-10). Prophylactic CMV prevention also is an important factor (11). The outcome of CMV infection is dependent on host and viral interaction (12). It seems that, intensity of immunosuppression affects the outcome (8). Treatment with mycophenolate mofetil (MMF) compared to azathioprine, has worse results (13-16). There are reports that mTOR inhibitors have synergic effect on ganciclovir and may have direct anti-CMV effects (7, 16-18). Asberg, *et al*, showed that lower total intensity of immunosuppressive therapy was associated with more effective early, but not overall, CMV DNAemia eradication by anti-CMV therapy. 

They also showed that MMF and tacrolimus therapy were associated with less chance of CMV recurrence (8). Haririan, *et al*. suggested that sirolimus as immunosuppressive regimen decreases the risk of CMV infection after kidney transplantation comparing to tacrolimus (19). Neyts *et al*. showed MMF is a potent enhancer of the anti-herpetic drugs. Also, topical therapy with acyclovir and MMF is an alternative to acyclovir resistant cutaneous herpetic lesions (20).Webster *et al*. performed a systematic review and meta-analysis of randomized trials and concluded that, using mTOR inhibitors instead of antimetabolites, acute rejection, CMV infection reduced (21). It was hypothesized that the lower the intensity of immunosuppression regimen, the less rate of conversion of CMV infection to CMV disease. We also investigated other possible risk factors effecting the outcome of disease.

## Method

**Study Design: **This was a cross-sectional study. Approval of this study was obtained from the Medical Ethics Committee (Approval no: IR.SBMU.RIGLD.REC.1400.007) of Shahid Beheshti University of Medial Sciences, Tehran, Iran. We retrospectively collected data from recipients who underwent orthotopic liver transplantation (OLT) between March 2014 and March 2016 and had positive PCR for CMV after transplantation in follow-up course. Of the 134 recipients, only sixty-six (7.8%) patients among the 842 liver transplantation were eligible. Of these, 50 (75.7%) patients had CMV infection and 16 (24.3%) had CMV disease. The variables below were recorded: age, sex, weight, blood group, etiology of liver failure, MELD score, cold ischemic time, warm ischemic time, intraoperative bleeding, immunosuppressive drugs and regimen, operative time, surgical complications, number of blood product transfusion, plasma CMV viral load, donor and recipient CMV IgG serostatus, rejections and corticosteroid pulse therapy. 

**Inclusion and Exclusion Criteria: **All recipients with clinical symptoms related to CMV with any serum copy and patients with serum PCR more than 500 copies/mL for CMV were included. Recipients without clinical symptoms and CMV viral load under 500 copies/mL were excluded from the study. Recipients on CMV prophylaxis regimen based on institutional protocol were excluded. 

**Definition of CMV disease and Infection: **Recipients with serum CMV more than 500 copies/ mL were considered as infected and patients with clinical CMV syndrome or tissue invasive disease documented with pathology, with any serum CMV count were labelled as CMV disease. 

**CMV Viral load Measurement: **Plasma viral loads for CMV were measured in all recipients as routine follow-up every three months after transplant and in case of clinical suspiciousness, the use of PCR technique. The amount of CMV viral load recorded was the amount at the beginning of treatment.

**Donor and recipient CMV serostatus: **Donor and recipient CMV IgG serostatus at the time of transplantation were registered from local hospital measurements. 

**CMV prophylaxis strategy:** Universal prophylaxis with valganciclovir was used in high-risk recipients. Patients treated with ATG, intubated, high MELD score, on CRRT were considered high risk for CMV infection.

**Immunosuppression Therapy: **Recipients received either methylprednisolone 1 gram for three days or thymoglobulin 1.5 mg/kg/day for a total dose of 6 mg/kg as induction immunosuppression therapy. Depending on the side effects of thymoglobulin, the dosage of drug was reduced. Cellcept, tacrolimus, cyclosporine, everolimus and prednisolone were used as maintenance therapy. Trough level of immunosuppressive medication mentioned in this study was checked whenever CMV PCR became positive.

**Acute Cellular Rejection: **Acute cellular rejection was documented based on pathology findings after clinical suspicious. 

**Statistical Analysis: **All data were analyzed with SPSS Version 21. Regression models were used for correlations. P- values are significant less than 0.05.

## Results

Among the 842 liver transplantations between March 2014 and March 2016, 66 (7.8%) patients were evaluated. Of these, 50 (76%) patients had CMV infection and 16 (24%) had CMV disease documented by biopsy or culture.

**Demographic and Baseline Characteristics:** Table 1 illustrates the demographic data of both groups. There was no correlation between blood group of donor or recipient and conversion rate. It also suggests that there is no correlation between etiology of liver failure, type of operation, cold ischemic time, warm ischemic time, recipient MELD score; and conversion rate of CMV infection to CMV disease. Based on data below, the p-value for correlation between recipient age, weight and conversion rate of CMV infection to CMV disease are 0.068 and 0.056.

**Table 1:** Demographic data and correlation of variables and CMV infection and disease group

Demographic Data n(%)	CMV Disease	CMV Infection	P-value
Recipient Blood Group			0.375
**A**	5(31.0)	18(36.0)	
**B**	3(19.0)	18(36.0)	
**AB**	1(6.0)	3(6.0)	
**O**	7(44.0)	11(4.0)	
**Donor Blood Group **			0.636
**A**	5(31.0)	19(38.0)	
**B**	3(19.0)	16(32.0)	
**AB**	1(6.0)	3(6.0)	
**O**	7(44.0)	12(24.0)	
Etiology of Liver Failure			0.594
**AIH**	4(25.0)	8(16.0)	
**PSC**	3(19.0)	4(0.8)	
**Overlap syndrome**	1(6.0)	2(4.0)	
**HBV**	3(19.0)	9(18.0)	
**Wilson**	1(6.0)	3(6.0)	
**Cryptogenic**	-	9(18.0)	
**NASH**	-	3(6.0)	
**Alcoholic**	-	2(4.0)	
**PBC**	1(6.0)	1(2.0)	
**Budd Chiari**	1(6.0)	2(4.0)	
**PFIC**	1(6.0)	1(2.0)	
**Acute liver failure**	-	1(2.0)	
**Cholangiocarcinoma**	1(6.0)	-	
**HCC**	-	1(2.0)	
**Pseudo papillary carcinoma**	-	1(2.0)	
**HBV&HCC**	-	3(6.0)	
MELD score			
**<=20**	8(50.0)	25(50.0)	>0.999
**>20**	8(50.0)	25(50.0)	
**Technique**			
**Piggy back**	12(75.0)	32(64.0)	0.547
**Standard**	4(25.0)	18(36.0)	
Biliary Reconstruction			
**Duct-to-Duct**	14(87.5)	45(90.0)	>0.999
**Roux-en-y**	2(12.5)	5(10.0)	
**CIT (min)**			
**<=360**	6	12	0.291
**>360**	10	38	
WIT (min)			
**<=35**	10	29	0.750
**>35**	6	21	
Recipient Age** (yr.)	34.0 ± 13.40	44.00 ± 12.30	0.068
Donor Age** (yr.)	34.00 ± 18.20	37.00 ± 15.60	0.509
Recipient Weight** (kg)	61.00 ± 12.30	71.00 ± 19.00	0.056
Donor Weight** (kg)	68.00 ± 17.60	72.00 ± 16.40	0.369
Hx. Of readmission			
**YES**	3 (19.0)	10(20.0)	
**No**	13 (81.0)	40(80.0)	

Abbreviations: AIH- Autoimmune Hepatitis; PSC- primary sclerosing cholangitis; HBV- Hepatitis B virus; NASH- Nonalcoholic steatohepatitis; PBC- primary biliary cirrhosis; PFIC- progressive familial intrahepatic cholangiopathy; HCC- Hepatocellular Carcinoma; MELD- Model for end stage liver disease; CIT- cold ischemic time; WIT-warm ischemic time.

**mean±SD

**Surgical Events and Complications: **Among the 66 patients, 4 (6.0%) had vascular complication including hepatic artery thrombosis and stenosis. Eighteen (27%) recipients needed reoperation for surgical complications mentioned below. Mean±SD volume of intraoperative bleeding was 970 cc±655cc. Mean±SD duration of operation was 230±41 min. As shown in table 2, the analysis showed no significant association between surgical complications, reoperation, intra operative bleeding, operation time, transfusion and conversion of infection to disease. Table 2 shows these correlations.

**Table 2:** Correlation between surgical complications, intraoperative bleeding, transfusion, operative time, conversion of infection to disease

Surgical events n(%)	CMV Disease	CMV Infection	P-value
Vascular Complication			
**YES**	1 (6.0)	3(6.0)	
**No**	15 (94.0)	47(94.0)	
Reoperation			
**YES**	6(37.5)	12(24.0)	0.340
**No**	10(62.5)	38(76.0)	
Complication after Tx			
Bleeding	3 (19.0)	10(20.0)	
**Liver necrosis**	-	1 (2.0)	
**Ureteral stones**	1 (6.0)		
**Bile Duct Stricture**	1 (6.0)		
**Incisional hernia**	1 (6.0)		
**No**	10(62.5)	39(78.0)	
Intraoperative** Bleeding (cc) **	970.00±655.00	1280±1050.00	0.332
Operation time ** (min)	230.00±40.00	240±59.00	0.562
Transfusion** (n)	1.25±1.65	2±2.20	0.222

Abbreviation: Tx, Transplantation.

**mean± SD

**Rejection: **Based on pathologic review, 11 (22%) recipients in CMV infection group and 8 (50%) recipients in CMV disease group had acute cellular rejection. In our center, we use IV methylprednisolone 1gr per day for 3 days as first line treatment in case of moderate or severe cellular rejection. If mild cellular rejection happens, we treat them with oral immunosuppressive drugs. All recipients received methylprednisolone for treatment of acute cellular rejection in this study. Nine (56%) more extra corticosteroid pulse therapy was needed in CMV disease group and 8 (16%) more pulse was needed based on clinical judgment. There was significant correlation between cellular rejection, extra corticosteroid pulse therapy; and conversion of CMV infection to CMV disease with p-value of 0.031 and 0.001. Table 3 shows the details. **Re-transplantation: **Two (4%) of CMV infected group were re-transplanted due to vascular complications in the first admission. In the diseased group, none of the recipients underwent re-transplantation. **Donor and Recipients CMV IgG serostatus: **All of our recipients were serologically positive for CMV IgG. Among the infected group, 47 (94%) donors were positive for plasma CMV IgG and 3 (6%) were negative. In diseased group, 10 (62.5%) donors were serologically positive and 6 (37.5%) were negative. Table 5 shows the correlation. There was significant correlation between donor plasma CMV IgG status and conversion of CMV infection to CMV disease. 

**Table 3:** Correlation of cellular rejection and extra pulse therapy; and conversion of CMV infection to CMV disease

Rejection and Therapy n(%)	CMV Disease	CMV Infection	P-value
Cellular rejection			
**YES**	8(50.0)	11(22.0)	0.031
**No**	8(50.0)	39(78.0)	
Extra corticosteroid pulse therapy^**^			
**YES**	9(56.3)	8(16.0)	0.001
**No**	7(43.8)	42(84.0)	

** For every pulse other than the first therapy

**Table 4:** Correlation of re-transplantation; and conversion of CMV infection to CMV disease

**Retransplantation n(%)**	**Disease**	**Infection**	**P-value**
YES	-	2(4.0)	>0.05
No	16(100.0)	48(96.0)	>0.05

**Table 5:** Correlation of donor and recipient CMV IgG serostatus; and conversion of CMV infection to CMV disease

Sero-status n (%)	Disease	Infection	P-value
Recipient CMV IgG			
**Positive**	16(100.0)	50(100.0)	>0.05
**Negative**	-	-	
Donor CMV IgG			
**Positive**	10(62.5)	47(94.0)	0.005
**Negative**	6(37.5)	3(6.0)	

**Table 6:** Correlation of immunosuppressive drugs and conversion of CMV infection to CMV disease

Immunosuppression	Disease	Infection	P-value
**FK Dose** ** ** (mgr.)**	4.30 ± 1.2	4.70 ± 7.3	0.133
**FK through level** ** ** (µg/ml)**	11.1 ± 5.7	8.0 ± 4.2	0.031
**Cellcept Dose** ** ** (gr.)**	2,250 ± 550.0	2,150.0± 550.0	0.355

Abbreviation: FK, Tacrolimus

**mean± SD

**Immunosuppression: **Our most efficient immunosuppressive drug is tacrolimus (Prograf, FK506). Mean±SD of tacrolimus through level in CMV disease group was 11.1±5.7 (µg/ml) and 3.1 (µg/ml) more than infected group (P=0.031). There was a significant association between FK trough level and conversion rate. There was no correlation between cellcept, thymoglobulin; and conversion rate. Table 6 shows the details.

## Discussion

Multiple factors have influence on severity of CMV disease and anti-viral therapy response rate. The immunosuppression intensity seems to have a major role. It is usually premised that, the more number or dose of immunosuppressive drugs used, the more chance of opportunistic infection post-transplant. This is also true for serum immunosuppressive trough level. The chance of viral complication after transplant is related to interaction between the host and viral response (12).Other factors are donor and recipient CMV IgG sero-status, anti-viral prophylaxis, rejection which are discussed individually. Prograf (tacrolimus) is the main immunosuppressant and a calcineurin inhibitor. Considering our results, we could conclude the severity of immunosuppression indicated by serum trough level affects conversion rate. Mycophenolate mofetil (cellcept) is an anti-metabolite immunosuppressant. Song *et al.* reported increased chance of CMV in recipients receiving MMF (15). We did not find any significant correlation between cellcept dose and conversion rate of CMV infection to disease. This is in contrast to data in literature. The reason for this finding is probably because cellcept was used with maximum dosage for all recipients. Wagner *et al.* did a systematic review comparing azathioprine (AZT) and MMF in kidney recipients. They concluded more invasive CMV disease in MMF group (13). None of our recipients received azathioprine, therefore we could not camper the effect of AZT and MMF individually. Harrian *et al. *reported the protective effect of sirolimus comparing to tacrolimus(19). Although, this was reported by others (17), we did not include m-TOR inhibitors in this study.

The precise effect of immunosuppressive drugs on immune system is not well known. Ahlenstiel-Grunow *et al. *conducted a multicenter randomized clinical trial arguing about the effect of immunosuppression on immune system (22). They believe that drug trough level monitoring is an incorrect estimation of patient’s immunosuppression intensity. They showed virus-specific T-cell (Tvis) is associated with viral replication and immunosuppression intensity. They argued that monitoring with Tvis improves graft function. This idea was reported earlier by Sester *et al. *(23, 24), Radaha *et al.*(25) and Gamadia et al. (26). Emphasizing on drug dose or serum trough level is insufficient for follow-up and leads to drug over dose (susceptibility to infections) or under-dose (acute cellular or humoral rejection) (22). Rejection is the net effect of under-dosing of immunosuppression. We found significant correlation between ACR and infection conversion rate. Obviously, treatment is extra-bolus corticosteroid therapy. We also found significant correlation between extra-corticosteroid therapies. Corticosteroids are the back-bone of immunosuppression and weaken immune response to viral infections. Status of CMV IgG level of donors and recipients are important. The highest risk is for positive donor organ transplanting to negative recipient who are not immunized to infection (D+/R-). We found significant correlation between donor CMV IgG serostatus and patients. CMV infection is very common among general population in the developing counties. All our recipients were positive serologically. Therefore, no association was detected. We had multiple limitations. This was a uni-center retrospective study. We premised the intensity of immunosuppression on individuals are estimated with total dosage or number of drugs used. Due to data presented above, this might not always be true. Therefore, future studies considering this issue is more accurate. In summary, intensity of immunosuppression including prograf through level, rejection, extra-corticosteroid pulse therapy, CMV donor IgG serostatus influence conversion of CMV infection to disease.
